# Modeling Healthcare Costs Attributable to Secondhand Smoke Exposure at Home among South Korean Children

**DOI:** 10.3390/ijerph17124496

**Published:** 2020-06-23

**Authors:** Jeewon Park, SeungJin Bae

**Affiliations:** College of Pharmacy, Ewha Womans University, Seoul 03760, Korea; jeewon941027@gmail.com

**Keywords:** secondhand smoke, SHS, Markov model

## Abstract

Children exposed to secondhand smoke (SHS) are at increased risk for disease. We sought to estimate the medical costs among Korean children who were exposed to SHS at home. A Markov model was developed, including five diseases (asthma, acute otitis media, acute bronchitis, pneumonia and sudden infant death syndrome) that were significantly associated with SHS in children based on a systematic review. The time horizon of the analysis was 20 years (from birth to adulthood), and the cycle length was 1 week. The direct healthcare costs were discounted annually at 5%. Univariate and probabilistic sensitivity analyses were conducted. The Markov model estimated the healthcare costs for 20 years as 659.61 USD per exposed child, an increase of approximately 30% compared to the cost per unexposed child (507.32 USD). Sensitivity analysis suggested that the younger the age of the exposure, the greater the incremental healthcare costs incurred, implying that infants and young children were especially vulnerable to the SHS exposure. Findings of this study could provide key baseline data for future economic evaluations on SHS control policies in South Korea.

## 1. Introduction

Secondhand smoke (SHS) contributes to premature deaths and diseases in both adults and children [1]. Worldwide, 40% of children, 33% of nonsmoker males, and 35% of nonsmoker females are exposed to SHS, resulting in more than 600,000 deaths in 2004 and accounting for 1% of the world’s mortality rate [2]. A recent study estimated the values of worldwide childhood disability-adjusted life years (DALYs) and deaths due to SHS exposure as 5.65 million (1.0% of all DALYs) and 63,823 (1.1% of all deaths), respectively [3]. In addition, children’s exposure to SHS causes more healthcare spending. Compared with SHS-nonexposed children, SHS-exposed children tend to visit the emergency room more frequently and have a significantly higher length of hospital stay, resulting in higher healthcare costs [4].

About 17.5% of adult Koreans smoke daily in 2017, which is slightly lower than the OECD (Organization for Economic Cooperation and Development) average (18%) [5]. Since 2014, South Korea has implemented various tobacco control policies. All restaurants are now designated nonsmoking areas and indoor smoking has been banned in stages. However, there is no policy in most countries, including Korea, to regulate SHS at home where children, who are particularly vulnerable to the adverse effects of SHS, spend most of their time [6]. In Korea, the SHS exposure rate in the home for 13–18 years of age is approximately 31.7% according to Youth Health Survey conducted in 2019 [7].

The World Health Organization (WHO) presented diseases attributable to SHS exposure, including low birth weight (LBW), sudden infant death syndrome (SIDS), lower respiratory tract infection (LRTI), otitis media (OM) (middle ear infection), and asthma [8]. Several studies have estimated the economic burden of SHS exposure due to those diseases using insurance claims data [9,10]. In children aged 0–17, the annual costs of exposure to SHS were 91.8 million USD in Minnesota and 261.1 million USD in North Carolina [9,10]. Most of the previous studies report short-term cost estimates using surveys in the US and claims data [4,9,10,11,12]. However, there is no medium- to long-term research on the disease burden of SHS in children who are most vulnerable to SHS exposure in the home. We employed a Markov model to forecast long-term economic and health effects of SHS exposure with currently available epidemiologic and economic data, since Markov model is useful when events are recursive like OM and asthma [13]. The purpose of this study was to estimate the economic burden of Korean children exposed to SHS in the home for 20 years. More specifically, we sought to project the direct healthcare costs attributable to SHS exposure among Korean children. Since this is the first study to estimate the long-term economic burden of SHS in children, the findings of our study can be useful when evaluating the economic impact of SHS control policy at home.

## 2. Materials and Methods

### 2.1. Model Overview

**Figure 1:**
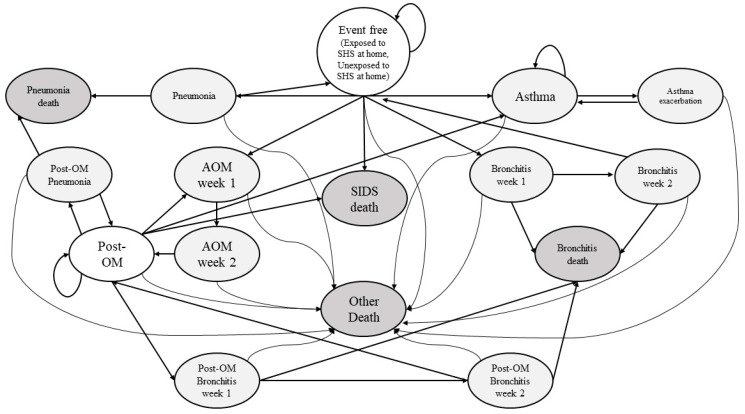
Health states and disease progression of Korean children who were exposed to SHS at home. SIDS, sudden infant death syndrome; AOM, acute otitis media; Post-OM, post-otitis media; SHS, secondhand smoke.

We modified a previously built Markov model to predict the economic burden of South Korean children who were exposed or unexposed to SHS in the home (Figure 1) [14]. The time horizon of the analysis was 20 years, from birth to adulthood. Since the study subject can smoke directly after becoming an adult, the time horizon of the model was limited to 20 years. The cycle length (transitions from one state to another) was one week, since the disease period of acute otitis media (AOM), acute bronchitis, and pneumonia is as short as one to three weeks [15,16,17,18]. We presented costs as an outcome, which were annually discounted at 5% based on the economic evaluation guidelines to reflect people’s positive time preference, namely, people prefer to benefit sooner than later [19,20]. TreeAge Pro 2019 software was used to simulate the model.

### 2.2. Target Population

A hypothetical cohort of Korean newborn infants with at least one of family member who was a current smoker was analyzed. All study populations were set to start from an ‘event free’ state at age of 0, with 50% of them being female. ‘Current smoker’ was defined as a person who smokes one or more cigarettes a day at home, based on previous studies about passive smoking, which is consistent with the definition of the daily smoker defined by WHO: someone who smokes any tobacco product at least once a day [21,22,23,24]. It was assumed that the target population did not smoke directly during the analysis period and were continuously exposed to SHS in the home until they reached adulthood (20 years old). Since the definition of SHS exposure in this study was limited to postnatal SHS exposure in the home, a low birth weight state due to maternal smoking in pregnant women was excluded from the model. Only exposure to SHS at home was considered, since children spend most of their time at home and there is a high possibility of continuous exposure; therefore, SHS in the home considered to be most important [6].

### 2.3. Input Variables

The increased risk of disease incidence due to SHS exposure was obtained through systematic review. Domestic data were preferred for input variables; however, in the absence of domestic data, international studies were used. The WHO guidelines suggest the types of disease and relative risk value of diseases recommended to be included for measurement of SHS burden in children [8]. However, considering that all the sources of the suggested values were literature published before 2006, we conducted an additional systematic literature review and searched for the appropriate values. After setting the search terms and PICO (population, intervention, comparison, outcomes) with reference to the previous studies, we searched through the PubMed, KISS, and KoreaMed databases [1,25]. We focused on meta-analyses or systematic review studies conducted in infants and children exposed to SHS. If the incidence of the disease is less than 10%, the odds ratio (OR) value can approximate the risk ratio (RR) value [26]. Diseases included in this study had an incidence less than 10%, so we used the OR value instead of the RR when the RR values were not available.

For the incidence rates, mortality rates, and transition probabilities, domestic data were preferably sourced. International studies were also referenced when domestic data were not available.

Only direct healthcare costs incurred by the NHI (National Health Insurance) and patients were included (sum of the government portion and patients cost sharing), and we sourced the cost estimates using the NHI claim database, since it provides the nationally representative estimates for general Korean population thanks to its extensive coverage [27,28]. Non-medical costs such as transportation costs, caregiver costs, and the cost of lost productivity were excluded. Healthcare costs from incidence-based cost studies conducted in South Korea were preferably sourced, since the treatment costs vary by country [19].

### 2.4. Markov Model

A Markov model was developed to simulate the natural history related to SHS exposure. Based on a systematic review, we identified five diseases whose risks were significantly increased with SHS exposure: SIDS, AOM, acute bronchitis, pneumonia and asthma [8]. All analysis subjects started from an event-free health state, and depending on the transition path and probability, they stayed in the current state, moved to another state or died. Since all health states were mutually exclusive and collectively exhaustive, it was assumed that no two or more of the diseases included in the model could occur simultaneously. Since a person who previously had AOM has a higher probability of AOM recurrence than a person who has not had AOM before, we built tunnel states to reflect a higher probability of AOM recurrence in the model [29]. Asthma was assumed to be a chronic disease; therefore, once a subject was diagnosed with asthma, they could not return to the event-free health state. For AOM and acute bronchitis, it was assumed that the symptoms persisted for 2 weeks, so the tunnel had two states each; pneumonia was set to one state based on the assumption that the symptoms persisted for a week [16,17,18]. The model was run until all children reached 20 years of age or died [30].

### 2.5. Sensitivity Analysis

Since this study was based on many assumptions, the robustness of the assumptions was investigated through sensitivity analysis. Univariate sensitivity analysis was conducted on disease OR values (95% confidence interval), discount rate (0%, 3%, 7.5%), exposure period (0–9 years old, 10–19 years old) and probability of asthma exacerbation (+/−20%). Probabilistic sensitivity analysis (PSA) was also conducted on the costs and disease OR values through second-order Monte Carlo simulations, which performed 10,000 iterative samples and visualized the result by a scatter plot. We applied a gamma distribution for costs and a log-normal distribution for OR values. Appropriate distributions for each variable were selected based on previous studies [31]. Table 1 shows the applied distribution of variables for probabilistic sensitivity analysis.

## 3. Results

### 3.1. Input Data

As a result of systematic review searching for increased risks due to SHS exposure, six different literatures were finally selected. Odds ratios applied in our model and referred literatures are shown in Table 2. Age-specific values were referred when possible.

For the baseline and disease-specific mortality rates, data from National Statistical Office were applied [30]. Since there was no domestic study on incidence rate of the included diseases, incidence rates of AOM, acute bronchitis, and asthma were sourced from international studies [29,32,33]. Incidence rate of pneumonia was estimated from the Korean NHI [34]. The annual incidence and mortality rates applied in the model are also summarized in Table 2. Values in Table 2 were put into the model in the form of weekly incidence and mortality rates through appropriate conversion formulas [13].

Weekly costs applied for the model and source literatures are summarized in Table 3. In case of asthma, cost data were obtained from a study conducted among Korean asthma patients using NHI claims data [39]. The cost per ER visit was applied for the ‘asthma exacerbation’ state [39]. In the case of the ‘acute bronchitis week 1’ state, the cost per event was applied since the treatment duration is usually consistent with the cycle length [40]. We assumed that no medical expenses were incurred at the second week of acute bronchitis and AOM, assuming that medical treatment was performed only once in the first week during the tunnel state [16]. For pneumonia and AOM, in the absence of previous economic burden studies available in Korea, Korean NHI database were referenced [34]. With reference to previous studies, the ICD-10 codes for pneumonia (J12, J13, J14, J15, J16, J18) and AOM (H65, H65.0, H65.1, H65.9, H66, H66.4, H66.9) were determined and used for data collection, and the cost per claim was applied for the ‘pneumonia’ and ‘AOM week 1′ states [41]. Costs were adjusted by the medical care component of the Consumer Price Index in Korea and then adjusted to 2018 US dollars; US $1 was equivalent to 1115.7 Korean won as of 2018 [42,43].

### 3.2. Base-Case Analysis

**Figure 2:**
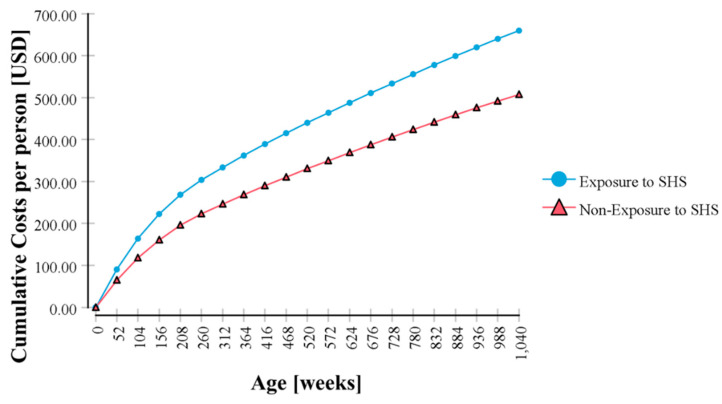
Cumulative healthcare costs for 20 years in Korean children who were exposed to secondhand smoke (SHS) at home compared with nonexposed children.

The Markov model estimated healthcare costs for 20 years as 659.61 USD per newborn baby in the SHS-exposed group, which was 30% higher (152.29 USD) than the estimated cost (507.32 USD) in the unexposed group (5% annual discount rate applied) (Figure 2).

### 3.3. One-Way Sensitivity Analysis

One-way sensitivity analysis showed that our model was robust in all variables except the odds ratio for pneumonia (Table 4). As a result of applying the value of the 95% confidence interval for pneumonia risk, the costs of the exposed group varied from 559.98 USD to 812.81 USD, which was a 10.38~60.22% increase in healthcare cost compared to the nonexposed group. According to the results of the one-way sensitivity analysis with different discount rates, as the discount rate increased, the rate of increase in healthcare cost of the exposed group to the unexposed was higher.

A sensitivity analysis was performed to determine whether the effects of SHS changed with exposure timing. One group was assumed to be exposed to SHS for the first 10 years (age of 0–9) and then not exposed from age 10 to 19. The other group was assumed to be not exposed to SHS for the first 10 years (age of 0–9) and then exposed to SHS for the next ten years (age 10–19). When exposed to SHS only during the first 10 years (0–9 years of age), the exposed group had a 25.99% increase in healthcare cost. On the other hand, when exposed to SHS only at ages 10–19, the cost increased by 3.02% (Table 4).

### 3.4. Probabilistic Sensitivity Analysis

Results of probabilistic sensitivity analysis are summarized in Table 5. The costs of SHS non-exposed group varied from 306.31 USD (minimum) to 751.07 USD (maximum) (145.20%). On the other hand, the costs varied more significantly in SHS exposed group, from 314.65 (minimum) to 1464.70 (maximum) (365.49%).

## 4. Discussion

The model predicted that in Korea, children and adolescents exposed to SHS in their home incurred an additional 152.29 USD (after 5% discount) or 218.45 USD (before discount) compared to the non-exposed group during the first 20 years of their life (until adulthood). According to the results of the one-way sensitivity analysis, higher healthcare costs were expected for the SHS-exposed group than for the nonexposed group, regardless of various assumptions.

To verify the validity of the model, we compared the results of our model with two studies that estimated the risk of pneumonia and AOM development due to SHS. Suzuki et al. (2009) reported that the risk of hospitalization due to pneumonia was OR = 1.55 in children exposed to SHS [14,44,45,46]. This is slightly higher than the OR = 1.41 obtained through the simulation model in the current study, but this is understandable considering the conservative approach of our study. According to Bentdal et al. (2007), the OR of AOM in children exposed to SHS was 1.3, which is somewhat smaller than our simulated result (OR = 1.5) [47]. However, Bentdal et al. (2007) identified the number of patients with AOM, not the number of episodes of AOM. Considering that AOM is a disease with frequent recurrence, our number is in line with Bentdal’s study. Therefore, the validity of our model is confirmed and the results of our study are reliable.

Several previous studies estimated the economic burden of diseases attributable to SHS exposure. According to Hill et al. (2008), SHS in the home increased the likelihood of emergency visits and hospitalizations due to respiratory symptoms, resulting in an increase in medical costs of 117 USD annually per child aged 0–4 in the United States [11]. Although the exact scope of the diseases included in the Hill et al. (2008) study and our study is different and difficult to compare, the annual costs of SHS exposure estimated by Hill et al. (2008) were 117 USD, whereas our study found that the cost was 152.29 USD for 20 years. The reason for the difference in amount is considered to be due to the conservative approach of our study. First, only five types of childhood disease, all of which had a clear association with SHS, were included in our model, whereas Hill et al. (2008) included all respiratory symptoms (ICD-9 code 460–519). Second, due to Korea’s National Health Insurance System, medical costs in South Korea are generally cheaper than those in the US. According to OECD data, in 2018, the annual medical expenditure as a percentage of GDP was 8.1% in South Korea and 16.9% in the US. In addition, South Korea and the US spent 3,192 USD and 10,586 USD per person for medical services in 2018, respectively, showing a large difference [48]. Differences in medical expenditure are also seen when comparing expenditure per episode for the same disease. According to a study conducted by Plescia et al. (2011), which estimated the healthcare costs of children and adolescents exposed to SHS in North Carolina through insurance claims data and MEPS (Medical Expenditure Panel Survey), the costs per case calculated from claims data were 515 USD for acute LRTI and 276 USD for OM [10]. On the other hand, the costs of those diseases applied to our model were significantly lower than the American counterpart (14.4 USD for acute bronchitis and 14.7 USD for OM, respectively). For these reasons, the estimated healthcare costs from previous studies show some differences from those estimated in this study. Given that the healthcare costs are relatively low in South Korea, the economic impact of SHS exposure in other developed countries could be even bigger.

Several assumptions were made in performing this study and there were methodological limitations. First, the time horizon of the model was limited to 20 years, from birth to the age of 20. Low birth weight due to maternal smoking during pregnancy and the risks from diseases related to SHS exposure in adulthood, such as lung cancer, stroke, myocardial infarction, and asthma, were not considered. However, according to a previous study that included those adult diseases in the Markov model, the predicted lifetime healthcare costs of SHS-exposed adult women were 600 USD higher than those of nonexposed adult women [14]. Additionally, this study included only direct medical expenditures in the analysis. However, according to Max et al. (2015), who estimated healthcare costs and cost of lost productivity resulting from premature death due to SHS exposure in children and adults, cost of lost productivity due to early deaths and poor quality of life accounted for approximately 33% of the total costs [12,49,50]. Therefore, not only the direct medical costs reported in this study but also the indirect costs due to SHS should not be overlooked.

Second, since this study is based on several assumptions by setting up a virtual cohort, it may not reflect all the characteristics of the real-world population. Unfortunately, confounders such as susceptibility, influenza, bad weather, and air pollutants were not controlled in this study, on the assumption that such confounders have the same effect on both SHS exposed and unexposed groups. Further study which incorporates those factors should be followed. In addition, we assumed that two or more diseases included in our model cannot occur concurrently, or the study population does not smoke until they become an adult, which might not reflect the natural history of the study population [51]. However, in order to prevent the model from becoming overly complicated, this study assumed that the diseases do not occur at the same time. Also, the effect of SHS exposure outside the home was excluded. Since the place where children spend the most time is inside the home and there is a high possibility of continuous exposure, SHS in the home was considered to be most important. However, a previous study reported that SHS exposure in public places significantly increases the risk of disease, so additional effect of SHS on healthcare costs in children may occur [52]. To sum up, our study provides conservative estimates based on various assumptions, thus the results should be interpreted with caution.

Third, to date, studies on the dose-response relationship of disease risk and SHS exposure at home are limited, and the definition of current smoker differs for each study. Our study crudely defined the exposure to SHS, therefore the concentration or amount of SHS in the home was not considered and the definition of smoker might look rather arbitrary. However, given that most of the sourced studies defined a current smoker as a person who smoked 1–19 cigarettes per day, our assumption reflects the definition of the previous studies [21,22,23,24].

Fourth, in the absence of cost studies in children, adult values were used. The cost of acute bronchitis used for the model was obtained from a study that included adult subjects, assuming that the costs would be similar for children and adults.

Finally, this study did not consider the effects of third-hand smoking (THS). Secondhand smoke includes both second-hand smoking, which is an exposure to cigarette smoke generated when a smoker exhales or burns cigarettes, and third-hand smoking, which is an exposure to residual nicotine absorbed by clothes, furniture, and skin. THS is expected to be more dangerous in children than in adults because children spend more time in the home, and children who crawl are more likely to touch objects that have absorbed harmful particles [53]. However, there is not enough research on the risk of specific diseases caused by THS, so this study did not include THS. Therefore, there is a possibility that the risk of actual SHS has been underestimated.

Despite these limitations, this study has significance in that it is the first study to estimate the long-term effects of SHS by constructing a simulation model for children and adolescents. Since the healthcare costs of children exposed to SHS have been approached with conservative assumptions, it is appropriate to consider the results in this study as the minimum costs consequences of SHS exposure. Additionally, comparison with the existing epidemiological data has confirmed the validity of our model and increased the reliability of our results.

Discussions of the adverse effects of SHS have been made mainly in terms of health outcomes. Our study provides the economic burden of SHS among children and adolescents who are most vulnerable to the adverse effects of SHS at home. Our study suggested that healthcare costs increased about 30% due to exposure to SHS at home, which could cause a significant burden to households and the NHI. These cost estimates can be vital baseline data to inform future economic evaluations on tobacco control policies.

## 5. Conclusions

SHS exposure at home had a significant negative economic impact on children and the result was consistent over a wide range of assumptions. In particular, our analysis showed that children exposed to SHS for the first 10 years (age of 0–9) incur considerable medical cost. Therefore, more aggressive preventive measures, such as SHS regulation at home, should be considered for the parents of the children younger than 10.

## Figures and Tables

**Table 1:** Distribution of variables for probabilistic sensitivity analysis.

Variables		Distribution
Odds ratio of disease	AOM first time	Lognormal distribution
	AOM recurrence	Lognormal distribution
	Acute bronchitis	Lognormal distribution
	Pneumonia	Lognormal distribution
	Asthma	Lognormal distribution
	SIDS	Lognormal distribution
Cost	AOM week 1	Gamma distribution
	Acute bronchitis week 1	Gamma distribution
	Pneumonia	Gamma distribution
	Asthma	Gamma distribution
	Asthma exacerbation	Gamma distribution

AOM, acute otitis media; SIDS, sudden infant death syndrome; Post-OM, post-otitis media.

**Table 2:** Annual incidence/mortality rate per disease state of patients who were not exposed to SHS and the odds ratio of morbidity related to SHS exposure used in the model.

Disease	Age	Incidence Rate	Ref	Age	Mortality Rate	Ref	Age	Odds Ratio	Ref
SIDS	0	0.0002	[30]	-	-	-	-	1.94 (1.55–2.43)	[35]
AOM first time	0–2	0.0717	[29]	-	-	-	-	1.32 (1.20–1.45)	[36]
	3–5	0.1588							
	6–19	0.041							
AOM recurrence	0–2	0.412	[29]	-	-	-	-	1.32 (1.14–1.52)	[1]
	3–5	0.381							
	6–19	0.267							
Acute Bronchitis	0	0.0048	[32]	0	0.000003	[30]	-	1.58 (1.27–1.98)	[37]
	1–4	0.0259		1–19	0				
	5–14	0.0113							
	15–19	0.0107							
Pneumonia	0–4	0.6920	[34]	0	0.000015	[30]	-	1.43 (0.93–2.21)	[37]
	5–9	0.1599		1–14	0.000001				
	10–14	0.0416		15–19	0.000002				
	15–19	0.0179							
Asthma	0–4	0.0234	[33]	-	-	-	0–2	1.14 (0.94–1.38)	[38]
	5–11	0.0111					3–4	1.21 (1.00–1.47)	
	12–19	0.0044					5–19	1.30 (1.04–1.62)	
Baseline mortality	-	-	-	0	0.002807	[30]	-	-	-
				1–4	0.00013				
				5–9	0.000076				
				10–14	0.000095				
				15–19	0.000216				

SHS, secondhand smoke; Ref, reference; SIDS, sudden infant death syndrome; AOM, acute otitis media.

**Table 3:** SHS-related weekly morbidity costs in 2018 US dollars used in the model.

State	Age	Weekly Healthcare Costs	Ref
Event free, Post-OM	0–19	0	-
AOM week 1	0–4	14.7	[34]
	5–9	15.0	
	10–14	17.2	
	15–19	21.4	
AOM week 2	0–19	0	-
(Post-OM) Bronchitis week 1	0–19	14.4	[40]
(Post-OM) Bronchitis week 2	0–19	0	-
(Post-OM) Pneumonia	0–4	104.2	[34]
	5–9	78.8	
	10–14	85.3	
	15–19	90.3	
Asthma	0–14	2.7	[39]
	15–19	3.1	
Asthma exacerbation	0–14	65.4	[39]
	15–19	99.2	

SHS, secondhand smoke; Ref, reference; AOM, acute otitis media; Post-OM, post-otitis media.

**Table 4:** Base-case and one-way sensitivity analysis results for Korean children who were exposed to SHS in the home compared with nonexposed children.

Parameters	SHS Exposure States	Direct Healthcare Costs (USD)	
		Total	Incremental (%)
Base-case			
	No	507.32	
	Yes	659.61	30.02
Discount rate			
0%	No	769.24	
	Yes	987.69	28.40
3%	No	591.46	
	Yes	765.19	29.37
7.50%	No	428.02	
	Yes	559.87	30.80
Exposure period			
0–9 years old	No	507.32	
	Yes	639.18	25.99
10–19 years old	No	507.32	
	Yes	522.64	3.02
Odds ratio for SIDS mortality			
Upper 95% CI	No	507.32	
	Yes	659.64	30.02
Lower 95% CI	No	507.32	
	Yes	659.58	30.01
Odds ratio for AOM morbidity			
Upper 95% CI	No	507.32	
	Yes	657.49	29.60
Lower 95% CI	No	507.32	
	Yes	661.69	30.43
Odds ratio for recurrent AOM morbidity			
Upper 95% CI	No	507.32	
	Yes	654.96	29.10
Lower 95% CI	No	507.32	
	Yes	664.77	31.04
Odds ratio for acute bronchitis morbidity			
Upper 95% CI	No	507.32	
	Yes	659.1	29.92
Lower 95% CI	No	507.32	
	Yes	660.27	30.15
Odds ratio for pneumonia morbidity			
Upper 95% CI	No	507.32	
	Yes	559.98	10.38
Lower 95% CI	No	507.32	
	Yes	812.81	60.22
Probability of asthma exacerbation			
+20%	No	492.52	
	Yes	641.85	30.32
−20%	No	522.03	
	Yes	677.28	29.74

SHS, secondhand smoke; Ref, reference; AOM, acute otitis media; Post-OM, post-otitis media.

**Table 5:** Summary of the probabilistic sensitivity analysis results.

Statistic	Costs	
	SHS Non-Exposed	SHS Exposed
Mean	491.77	648.49
Standard deviation	58.99	141.17
Minimum	306.31	314.65
2.5%	384.08	430.40
10%	417.72	487.44
Median	489.97	629.75
90%	567.82	833.63
97.5%	614.76	976.57
Maximum	751.07	1464.70
